# Ethical challenges in research on post-abortion care with adolescents: experiences of researchers in Zambia

**DOI:** 10.1080/11287462.2018.1528657

**Published:** 2018-10-03

**Authors:** Joseph M. Zulu, Joseph Ali, Kristina Hallez, Nancy E. Kass, Charles Michelo, Adnan A. Hyder

**Affiliations:** a School of Public Health, University of Zambia, Lusaka, Zambia; b Department of International Health, Johns Hopkins Bloomberg School of Public Health, Baltimore, MD, USA; c Johns Hopkins Berman Institute of Bioethics, Baltimore, MD, USA; d Department of Health Policy and Management, Johns Hopkins Bloomberg School of Public Health, Baltimore, MD, USA

**Keywords:** Post-abortion care, research ethics, adolescents, ethics guidance, reproductive ethics

## Abstract

Post-abortion care (PAC) research is increasingly being conducted in low- and middle-income countries (LMICs) to help reduce the high burden of unsafe abortion. This study aims to help address the evidence gap about ethical challenges that researchers in LMICs face when carrying out PAC research with adolescents. Employing an explorative qualitative approach, the study identified several ethics challenges encountered by PAC researchers in Zambia, including those associated with seeking ethics and regulatory approvals at institutional and national levels. Persistent stigma around abortion and community perceptions that PAC studies encourage adolescents to seek abortion affected adolescents’ right to exercise their autonomy and to make decisions as well as exposed adolescents to social stigmatization risks. Challenges with recruitment was reported to result in abandoning of studies, thereby undermining development of PAC services that are more responsive to adolescent needs. Training needs identified included knowledge of best practices for conducting and disseminating PAC research. Strategies for addressing the ethical challenges included trust building and using less value-laden terminology when seeking permission and consent. It is essential to the future of PAC research in Zambia and globally that these important challenges be addressed through the development of comprehensive ethics guidance.

## Introduction

Unsafe abortions among adolescents is a major health challenge for health systems in low- and middle-income countries (LIMCs) (Cresswell et al., 2016, Hanschmidt, Linde, Hilbert, Riedel-Heller, & Kersting, 2016). About 13% of maternal deaths globally result from unsafe abortions, and the proportion of maternal deaths from unsafe abortion in some regions of sub-Saharan Africa is as high as 30% (Rogo, Oucho, & Mwalali, 2006). It is estimated that over 19 million cases of unsafe abortion occur around the word annually, and these are the cause of as many as 68,000 annual deaths (Grimes et al., 2006). Further, one quarter of all unsafe abortions in Africa are administered to adolescents (Grimes et al., 2006).

Zambia is a lower middle-income country located in southern Africa (Central Statistical Office (CSO) [Zambia], Ministry of Health (MOH) [Zambia] and II, 2014). About 60% of the population lives below the internationally recognized poverty line, i.e. on less than 1.90 USD a day. Almost 53% of the total 14 million inhabitants are under the age of 18 years (Central Statistical Office (CSO) [Zambia], Ministry of Health (MOH) [Zambia] and II, 2014) and as many as 31% of those aged 20–24 at the time they were interviewed were married before their 18th birthday (Central Statistical Office (CSO) [Zambia], Ministry of Health (MOH) [Zambia] and II, 2014). The country faces numerous health systems challenges (Central Statistical Office (CSO) [Zambia], Ministry of Health (MOH) [Zambia] and II, 2014). Maternal mortality is high (398/100,000 live births), with about 30% of these deaths due to unsafe abortion (Cresswell et al., 2016) and it has been estimated that approximately 80% of women taken to health facilities for abortion-related complications were adolescents (Muzira & Njelesani, 2013). Factors that influence unsafe abortion amongst adolescents include the stigmatization often associated with adolescent pregnancies and complex regulations regarding abortion (Sedgh & Hussain, 2007). Additional factors include the advice of trusted others, perceptions of risk, delays in seeking health care and the economic cost of seeking care (Coast & Murray, 2016). Social cultural factors, such as religious values, also influence access to reproductive health services including abortion (Zulu, Lisulo, et al., 2014).

Post-abortion care (PAC) comprises of different interventions, including community partnerships, maternal healthcare, individual counselling, and family planning, all designed to help reduce and respond to post-abortion-related complications (Corbett & Turner, 2003; Speckhard & Rue, 1992). Increases in sexual and reproductive health (SRH) challenges arising from unsafe abortion have contributed to an increase in abortion research globally (Cresswell et al., 2016; Gipson, Becker, Mishtal, & Norris, 2011; Sedgh, Henshaw, Singh, Åhman, & Shah, 2007). This research includes studies estimating the abortion rate, documenting experiences in seeking abortion services, and examining the uptake and quality of abortion services.

A recent scoping review on ethical issues faced by PAC researchers and other studies suggest that PAC researchers often face a range of ethical challenges in conducting research, including difficulties in recruiting study participants and disseminating study results, particularly when studies take place in LMICs and with adolescents (Adler et al., 1990; Bradley, Sikazwe, & Healy, 1991; Geary, Gebreselassie, Awah, & Pearson, 2012; Kinaro, Mohamed Ali, Schlangen, & Mack, 2009; Macleod & Hansjee, 2013; Sedgh et al., 2007). Specific challenges include difficulties in effectively protecting confidentiality, managing negative effects of interventions, creating a non-prejudicial atmosphere for research and managing emotional issues among adolescents (Adler et al., 1990; Bradley et al., 1991; Geary et al., 2012; Kinaro et al., 2009; Macleod & Hansjee, 2013; Sedgh et al., 2007; Zulu et al., 2018).

While such obstacles have been reported, the above-mentioned scoping review showed that studies aimed at documenting the ethical issues faced by PAC researchers in LMICs, and how they have been addressed in practice, are lacking (Zulu et al., 2018). The review recommended the need for further research to clarify the challenges and support the development of formal guidance in this area (Zulu et al., 2018). This study sought to address this gap by empirically exploring how researchers experienced and resolved ethical challenges encountered while conducting research on PAC with adolescents in Zambia.

## Methods

### Zambian context

Zambia has ratified major international and regional conventions and protocols on the rights of women, such as the Maputo Protocol (African Union, 2003). Abortion in Zambia is currently permitted by law under limited circumstances under the 1972 Termination of Pregnancy Act. Grounds for abortion include the need to save the life of the woman, to preserve physical and mental health of the mother, foetal impairment and economic or social reasons including minority (under 18 years old). Three medical doctors including one specialist can decide that abortion can be legally performed in specialized hospitals. However, the law has not advanced safe abortion services; in fact, access to safe abortion has been made nearly impossible particularly for young, poor, and rural girls through a requirement that the written consent of three medical doctors be obtained.

### Design and sampling

This study used an exploratory phenomenological study design to collect data on the ethical challenges faced by researchers studying PAC involving adolescents in Zambia (Guest & MacQueen, 2012). The phenomenological design was relevant as it focuses on individuals’ lived experiences, feelings and perceptions (Guest & MacQueen, 2012; Zulu, Kinsman, Michelo, & Hurtig, 2014). Using this design, we sought to discover, analyse, clarify and seek patterns of the phenomenon of ethical challenges conducting PAC involving adolescents in Zambia as experienced by the researchers as well as how they have sought to resolve them.

The study sample consisted of researchers involved in research on adolescent PAC at a large public university and at non-governmental organizations (NGOs) conducting adolescent health research in Zambia. Purposive and snowball sampling were used to identify study participants. These sampling approaches supported identification of diverse group of researchers with relevant experiences. The study started with purposive sampling as the aim was to first approach the researchers that fitted the purpose of the study. After all the researchers that we knew had been approached, the study employed snowball sampling which involved asking the researchers that we had interviewed for details of others researchers that conduct PAC research (Teddlie & Yu, 2007).

### Data collection

Primary data were collected through in-depth interviews, using an interview guide. The guide was developed with input from four bioethics and public health researchers from Johns Hopkins University, two Zambian bioethics experts and two researchers from Mulago National Referral and Teaching Hospital in Uganda and University of Botswana. The tool was pretested with researchers at the University Teaching Hospital in Lusaka, Zambia. Two members of the study team with experience in qualitative research collected the data. Study interviews lasted approximately 30 minutes and explored issues researchers encountered when conducting PAC research. In addition, they attempted to identify how researchers handled the identified ethical issues, probing about any sources of guidance. Interviews further inquired about ethical challenges with which researchers were familiar (e.g. relayed by other PAC researchers), even if they had not experienced these challenges themselves, and how they believed these challenges were or were not being addressed. All the interviews were conducted in English and audio-recorded. We also took notes during the interviews. Given the potentially sensitive nature of the subject matter, careful attention was given to maintaining confidentiality of data by not including identifiers of the respondents in the results. After every interview, we reviewed the notes and audio recording and discussed the process. This enabled us to reflect on the major issues that surfaced, identify complex issues that required further clarification, and pose additional questions during the subsequent interviews.

### Data analysis

Interview data were transcribed by a research assistant and reviewed by the first author. The first author and research assistant then read the transcripts, identified emergent codes and compiled them within a codebook that was reviewed by other authors. Upon author agreement, the first author coded the transcripts using NVIVO version 7 (QSR Australia) and shared with a research assistant for verification. Using a thematic analysis approach, codes were merged into categories and translated into themes (Braun & Clarke, 2006). All authors took several turns reviewing the manuscript to develop the final themes, focusing on the ethical issues researchers encountered when conducting PAC research (from planning to dissemination of the research results) and how they handled the challenges. Our findings were organized as such in order to increase their accessibility for researchers, regulators and others engaged in the practice and oversight of research. Table 1 shows the prevalent themes, ethical issues and related strategies used by researchers in addressing the ethical issues.

**Table 1.** Themes, ethics issues, and related strategies.

Theme	Ethics issues	Related strategies
Regulatory and policy challenges	Lack of policy on abortion research Ministry of Health officials not often discussing abortion issues in meetings Limited knowledge of abortion law in Zambia	
Difficulties with ethical review and approval	Limited knowledge of the legal provisions by some ethics committee members Requirement for researchers to have specialized health personnel on their studies Some staff at Ministry of Health being anti-abortion	
Challenges with recruitment	Recruitment based on patient registers Perception that PAC studies encourage young girls to seek abortion The taboo of discussing abortion Fear of being reported to the police Stigmatization Lack of awareness of the law on abortion by study participants	Trust building Recruitment of participants using health workers Substituting the word abortion with fertility control or reproductive health when seeking consent
Challenges with data collection	Difficulties in speaking about personal experiences Incomplete information on PAC from files	Holding interviews far from the health facility Use of female data collectors Substituting the word abortion with fertility control or reproductive health when collecting data
Research reporting	Difficulties in deciding how to report findings Difficulties in deciding avenues for sharing reports Concerns raised regarding masking study sites and providers (acceptable levels of transparency in describing research methods)	

## Results

### Respondent characteristics

Interviews were conducted with 12 PAC researchers including seven females and five males. One researcher requested that his/her data not be included in the final analysis and s(he) did not provide any reasons for the decision. Of the 12 researchers, 4 were involved in clinical research and 8 conducted behavioural research.

### Ethical challenges encountered by PAC researchers

Findings have been grouped into the following themes, further detailed below: regulatory and policy challenges, challenges with Institutional Review Board (IRB) processes, challenges with consent and recruiting adolescents, challenges with data collection, and challenges reporting PAC study findings. We first review these identified challenges individually, then discuss potential strategies that were suggested to address some of these challenges.

#### Regulatory and policy challenges

More than half of the researchers reported that there were several policy documents in Zambia related to abortion or reproductive health, but there was also a lack of policy on abortion research, particularly post-abortion care research with adolescents. These documents included the 1972 Termination of Pregnancy Act (Ministry of Justice, 1994), 2000 National Reproductive Health Policy (Ministry of Health, 2000), 2009 Health Professions Act (The Health Professions Council of Zambia, 2009), and the 2011–2015 Adolescent Health Strategic Plan. The absence of abortion issues in the 2000 National Health Policy was cited as a specific information gap. The National Reproductive Health Policy (under the National Health Policy) was also viewed as being silent on abortion matters. One interviewee noted:

The National Health Policy and Reproductive Health Policy are very silent when it comes to safe abortion, and their focus was mainly family planning. So, it is this that makes it hard to do abortion research because there are few general policies about abortion.

Although the abortion act permits abortion under special conditions, most researchers reported that its relevance is undermined by the lack of public awareness in Zambia about abortion research. A few researchers stated that Ministry of Health officials do not often discuss issues relating to abortion in meetings. According to these respondents, the limited discussion of abortion by staff at the Ministry of Health makes it hard to do research on abortion because, for example, some officials are not comfortable discussing PAC research results during dissemination workshops. In some cases, it is also difficult to interview officials regarding PAC issues as they were uncomfortable to discuss abortion issues. Most researchers stated that officials often mask the term abortion because it is not well received by many, including national health policy-makers and implementers. One researcher reported the term is often masked within the fertility control concept:

Even in meetings with the Ministry, people will discuss other issues and embed abortion in other issues.

Despite Zambia having one of the most liberal laws on abortion in Africa, all researchers reported that it was still difficult to conduct abortion-related studies because most community members and health workers lack adequate knowledge of the abortion law in Zambia. Lack of knowledge of the law made some community members and health workers want to disassociate themselves from any PAC research as they viewed such studies as contravening the law. This situation can undermine the potential for benefits of PAC research to be realized in some communities. One interviewee reported that most people including some health workers falsely believe that abortion is illegal in all cases:

Most health workers and the communities are ignorant of the abortion act which allows abortion under special circumstances such as if there’s risk to the health of women.

#### Challenges with IRB review and approval processes

Challenges with IRB processes also affected the conduct of PAC research. There were variations regarding researchers’ experiences with the process of seeking ethics approval for PAC research. While a small number of researchers had only a few challenges in seeking approval, others reported greater difficulties with the approval process. They attributed most challenges to limited knowledge of the legal provisions in Zambia by some ethics committee members. An additional challenge was the ethics committee requirement for social science researchers to recruit specialized clinical health personnel to support their social/behavioural research. A few researchers stated that finding specialized medical doctors was not easy due to limited human resources for health services in the country:

Sometimes the committee demands that we include a gynaecologist as a key person for the study, this becomes a challenge.

Meanwhile, some of the researchers who did not experience any challenges with the local ethics committee had challenges when the study went for review with the Ministry of Health, a requirement in the country for all studies. They attributed their difficulties in obtaining approval from the Ministry of Health to cultural values and beliefs regarding abortion among the members of staff in the Ministry, leading Ministry officials to be hesitant to allow researchers to study the practice.

I have no challenges with the IRB [Institutional Review Board]. Some decisions in the Ministry of Health are driven by the personal, religious or even cultural values of the policy makers. So sometimes these tend to be stumbling blocks, it’s sometimes frustrating in an era where policy should be driven by evidence.

A few researchers reported that these regulatory, institutional and policy challenges made them abandon PAC studies. They complained that abandoning studies made it difficult for them to contribute evidence-based information that could have helped make PAC services more responsive to adolescent-specific needs and challenges.

#### Challenges with recruitment and consent

Most of the PAC researchers indicated that they identified potential adolescent study participants using various strategies, including review of patient registers from hospitals at which adolescents had sought abortion related services. Some participants were also recruited from the community through a snowball sampling approach. All researchers stated that participant recruitment was challenging in part due to several ethical complexities outlined below. Challenges in recruiting participants for PAC research was suggested to be a significant reason why PAC has not advanced further in Zambia.

Seeking community acceptance of a PAC study involving adolescents was generally classified as difficult by all researchers. Such studies were deemed challenging due to the word “abortion” within the concept of post-abortion care. Researchers said that when they study things other than abortion it was easier to work with adolescents, but when they study abortion in adolescents they faced problems. The researchers who experienced challenges noted that the community in which the researchers conducted their studies perceived researchers as having a hidden agenda of promoting abortion among adolescents:

Research on abortion would be perceived to be a way … to begin encouraging young girls to seek abortion.

Recruitment and consent was also impacted by the taboo of discussing abortion. More than half of the researchers reported that community members often refuse to participate in PAC studies following consent disclosures due to social and cultural concerns. They suggested that some community members including adolescent girls and their guardians, preferred that the issue of abortion not be discussed as they considered it a taboo:

In most instances, once an abortion has taken place most guardians would prefer that it is not referred to again and even when the girls are identified as having aborted before, they (guardians) will refuse the adolescents to participate in the study.

Researchers’ further reported that fear of being reported to the police affected voluntary participation in their studies. All researchers faced enrolment difficulties because some adolescents fear being reported to the police. Researchers stated that even when they went to health facilities, they had difficulty identifying those who have had abortions because often adolescents did not want to be identified. The researchers reported that the fear was more pronounced in cases involving abortions that were not permitted under law. Not only did adolescents not want to be identified as having abortions, they also often did not want to implicate any health workers involved, who are often paid discretely for the service. One interviewee noted:

It’s difficult to enrol such adolescents in a study and adolescents rarely give information of a previous abortion done. So, you have to ensure you assure them you are not taking them to the police. It’s not as fast as other research, for example, on deliveries.

#### Challenges with data collection

The social, mental and physical effects associated with social stigmatization that may arise as result of participating in PAC research limited the extent of adolescent participation in many studies. All researchers reported that some participants fear that researchers may publicize their names and that this may lead to social stigma. Researchers indicated that the service seekers they interviewed stated that both service seekers and providers face stigma. Researchers further reported that studying and even just talking about abortion attracted negative perception from others. It was reported that even some civil society organizations that deal with sexual and reproductive rights, gender inequality and general health as well as human rights issues do not want to discuss abortion. For example, during promulgation of the Termination of Pregnancy Act in 2015, it was suggested that an unwillingness of people from different civil society organizations to talk about abortion affected the review process:

The consultant hired to review the Termination of Pregnancy Act only managed to interview a few civil society organisations because most were hesitant to talk about abortion. It’s easier to mask it under something general.

The fear of stigmatization and inadequate knowledge of applicable laws likely made it hard to collect information from adolescents about their experiences with abortion and PAC. For example, after noticing that some adolescents did not want to respond to questions about whether or not they had had an abortion, researchers often changed their questions to focus indirectly on adolescents’ perceptions and experiences. One researcher stated

The idea was to ask if one ha[d] ever had an abortion but we didn’t get the responses we needed so we would ask if they knew of anyone who had an abortion. We further changed [the] tool and just asked history of pregnancies to see if it resulted in a live-birth or otherwise and hoped that people would talk about abortion.

#### Challenges reporting PAC study findings

Concerns about effects of stigmatization also undermined reporting of PAC study findings. Different views were expressed by researchers related to sharing study findings. A majority of those interviewed experienced challenges in reporting PAC study findings. Most of the researchers noted that they often had difficulty determining how to report findings and deciding which avenues they should use to share their reports. For example, some found it difficult to strike a balance between being specific in reporting methods or findings and avoiding (potentially) exposing individuals or communities to legal or social risk. Other researchers reported concerns that by disseminating PAC findings, they may be labelled as being complicit in illegal or unsafe abortion. Additionally, researchers noted difficulty in reaching crucial service providers and policy makers who have made it known that they feel uncomfortable attending dissemination events because they fear being associated with abortion. Finally, there were concerns raised regarding masking study sites and providers, in addition to research funders, raising questions around acceptable levels of transparency in describing research methods. As one researcher explained:

Some donors don’t want to be publicly known as having funded or advanced a certain agenda. So, you may need to disseminate without disclosing your funder or the specific location for the sites were data was collected from.

## Potential strategies for addressing some of the identified ethical challenges

Respondents also described potential strategies for addressing some of the identified ethical challenges. These include training, trust building, recruiting participants using health workers, use of alternative language, holding interviews far from the health facility, and use of female data collectors. In what follows, we discuss each of these strategies.

### Training

Interviewees raised several training needs and opportunities to help address research capacity and awareness among key stakeholders. Research capacity in Zambia related to abortion was reported to be quite low, affecting the ability to conduct quality research to inform efforts to decrease unsafe abortions. One interviewee stated:

Zambia’s current capacity to carry out post abortion research is very low because [we] have poor research capacity, poor funding and poor proposals and also the fear to deal with controversial and emotive topics, as well as religious perspectives.

The researchers suggested that there is a need to train PAC researchers on optimal methods for conducting PAC research, taking into consideration that it relates to a highly sensitive matter in Zambia. One recommended training topic was how to engage key stakeholders or “gatekeepers” such as parents and guardians without compromising the consent process. Other researchers proposed the need for training on disseminating or communicating sensitive information to different audiences while avoiding risk to informants or stakeholders.

### Trust building

Researchers reported that they attempted to address ethical challenges with recruitment of study participants through trust-building strategies. One suggested way of developing trust was by having researchers talk directly with parents, guardians or other third parties associated with adolescents about the aims of the study, in general terms. Researchers further addressed recruitment challenges by developing partnerships with people who had positive views on abortion:

A way around it for us has been to build allies with people that are receptive to that study.

### Recruiting participants using health workers and alternative language

All researchers reported that they sometimes hire health workers and train them in data collection procedures, including seeking consent as a strategy to address difficulties in the recruitment process. Some researchers mentioned involving health workers because they knew they were respected and trusted by the community:

We didn’t have much trouble recruiting the clients because we work through the providers themselves. So, we trained the service providers and recruited through them and as a result we didn’t face any challenges. Often we went beyond our sample size.

Other researchers reported using a broader and more socially acceptable concept of reproductive health when recruiting and seeking consent and assent, as opposed to only mentioning abortion. When approaching parents, they did not disclose that their child had an abortion and would instead speak in general terms. For example, some researchers addressed barriers in the recruitment process by emphasizing the need to reduce unsafe SRH practices. Other researchers stated that they would like to speak to the adolescent about SRH including issues of abortion:

It was much easier to recruit because we did not explicitly say we were doing a study on abortion but a study looking at women’s reproductive health including abortion.

### Holding interviews far from the health facility

Other researchers opted to hold interviews at venues outside of health facilities to increase participant comfort and privacy. Respondents were not comfortable being interviewed at health facilities for fear of security officers or encountering people who they may be familiar with. One researcher noted:

[T]he adolescents did not want to meet at the clinic because they felt they were going to be arrested. They wanted to be certain that this was not a trap.

### Female data collectors

It was reported that working solely with young female data collectors to conduct studies with adolescents also improved the research process. Most of the researchers suggested that employing female data collectors helped make study participants feel more comfortable and able to express themselves:

To ensure that participants were comfortable, our study recruited young females to conduct the interviews and this seemed to provide the adolescents with a sense of confidence.

## Discussion

The study identified multiple ethical challenges faced by researchers who conduct studies on PAC with adolescents in Zambia, and documented suggested strategies used by them for responding to some of the challenges. Researchers working in this area reported several ethical challenges, including those associated with negotiating policy dynamics, seeking and securing ethical approvals, recruiting study participants, collecting data, and reporting data. Other studies have shown that conducting PAC research with adolescents raises particular challenges, underscoring both the need for more research with that group, but also more ethical guidance (Gipson et al., 2011; Hess, 2006; Ringheim, 1999; Söderberg, Andersson, Janzon, & Sjöberg, 1998).

One major ethical challenge during the recruitment phase was securing valid informed consent, a concern also noted in other studies (Gipson et al., 2011; Hess, 2006; Ringheim, 1999; Söderberg et al., 1998; Zulu et al., 2018). Several respondents reported that it was difficult to effectively recruit adolescents into PAC studies and carry out the consent and assent process mainly due to the taboo of discussing abortion, inadequate knowledge of the law by the community, and the stigma and discrimination associated with abortion. These barriers limit the extent to which adolescents can freely access information and make informed decision about participation in abortion related studies. Being inadequately informed can undermine individual autonomy and the capacity to make decisions without undue interference from others (Marshall, 2006). The inability to make informed decision may also deny these adolescents the benefits associated with such studies such as free medical care, check-ups, and information on sexual and reproductive health provided through the study (Childress et al., 2002; Tindana, Kass, & Akweongo, 2006).

Some researchers attempted to minimize social stigma associated with participation by substituting the word ‘abortion’ with more socially acceptable concepts, such “fertility control”, when recruiting participants and seeking consent. Other researchers and scholars have suggested that similar masking efforts during recruitment and consent can be appropriate under circumstances where there is a need to reduce risk for participants (Heisig, Shedden-Mora, Hidalgo, & Nestoriuc, 2015). Framing information during the consent process is recommended when doing so is likely to protect the wellbeing of participants (Heisig et al., 2015). When masking, it is important to be clear that no information is being withheld per se, but that it is being presented in a broad rather than specific way. This method compromises specificity for the sake of reducing risks and stigma. Thus, including abortion in a list of topics to be discussed allows both a reduction of stigma and the specificity one might desire. For example, saying that the study will explore many topics in sexual and reproductive health including contraception, sexual diseases, and abortion, may reduce stigma while still being transparent.

The International Ethical Guidelines for Biomedical Research Involving Human Subjects states that “deception is not permissible, however, in cases in which the deception itself would disguise the possibility of the subject being exposed to more than minimal risk, the investigators must demonstrate to an ethical review committee that no other research method would suffice” (Council for International Organizations of Medical Sciences & World Health Organization, 2002).

Researchers should not mask in cases where researchers’ decisions may oppose what study participants want; when researchers anticipate that problems may arise due to participation in the study; when the researchers know how the problems can be prevented; and when researchers are able to “warn” patients or study participants in advance of effects of their actions (Edmund Howe, 2008).

Other researchers tried to improve the recruitment process by engaging health workers and females to recruit adolescents as well as by holding interviews outside of health facilities. These strategies were adopted to promote privacy and confidentiality as well as respect for adolescents (Beauchamp & Childress, 2001; Kass, 2001). However, engaging health workers, if not well managed, may be problematic as the presence of health workers may trigger undue pressure for adolescents to participate in research due to differences in social status. Undue pressure may compromise adolescents’ autonomy, a view in line with other studies that have explored the role of social positions and power inequalities in consent processes (Marshall, 2006; Tindana et al., 2006). The International Ethical Guidelines for Biomedical Research Involving Human Subjects stresses that physicians and other health care professionals should assure the participants that whether or not they decide to participate in the study, the therapeutic relationship will not be disturbed. Further, guidelines outline that the ethics committee should consider the possibility of having a neutral third party to manage the recruitment (Council for International Organizations of Medical Sciences & World Health Organization, 2002).

Some researchers attempted to address the challenge of recruitment by checking through medical records at a hospital for adolescents who accessed PAC, and then following up such adolescents. We note that while it may be a challenge to recruit adolescents, it should not be acceptable for researchers to go to clinics and look through records of who has had an abortion; such practices undermine respect and privacy of the adolescents (Council for International Organizations of Medical Sciences & World Health Organization, 2002). Furthermore, such practices may trigger emotional and psychological harm or distress among adolescents who did not expect their records to be accessed by researchers without their consent (Council for International Organizations of Medical Sciences & World Health Organization, 2002; Ringheim, 1999).

As outlined earlier, the described ethics challenges also affected the quality and impact of data from adolescent PAC studies. For example, risks associated with stigmatization undermined participation in studies among adolescents, a finding that resonates with a recent scoping review on ethics challenges related to research involving PAC (Zulu et al., 2018). Researchers sometimes changed their questions to focus indirectly on adolescents’ perceptions and experiences, or completely abandoned their studies. Abandoning studies reduces the potential for research to contribute to the improvement of PAC services, thereby undermining the potential for benefit to individuals and communities (The National Commission for the Protection of Human Subjects of Biomedical and Behavioural Research, 1979).

Even where participants are successfully recruited, and data are collected, a range of difficulties associated with disseminating potentially sensitive PAC research findings emerged. These included difficulties in determining how to report findings to maximize uptake and impact, while minimizing potential for harm to communities or individuals. Should these challenges continue, it will remain difficult for research to positively influence policy and practice to address unsafe abortions for adolescents in Zambia.

To address many of the ethical challenges experienced by researchers who conduct PAC research with adolescents, it may be appropriate to adopt an “ecological approach” as the barriers to PAC research in Zambia exist at various levels. This approach stresses developing strategies that simultaneously address issues at individual, family, community, societal and institutional levels (Kumwenda, Nzala, & Zulu, 2017; Svanemyr, Amin, Robles, & Greene, 2015; Zulu et al., 2018; Zulu, Lisulo, et al., 2014; Zulu, Michelo, et al., 2014). Using the ecological approach would, for example, entail developing effective communication strategies that target dissemination of information regarding the grounds for legal abortion to adolescents, families, communities, IRBs and institutions more generally, such as the Ministry of Health. For this process to be constructive and effective, it is important that strategies for disseminating information be formulated in partnership with public institutions, the community and civil society organizations. Awareness of the legal context may help reduce stigma and fears associated with accessing PAC services as well as participating in PAC research. At the level of the researcher, the process should go beyond dissemination to include training on acceptable strategies for collecting and disseminating sensitive information. Further, there is need for community engagement and discussion regarding the importance of PAC research, the ethical oversight that it receives, and the nature of the legal framework related to abortion in Zambia. It may be helpful for the community to provide input into the conduct of PAC research as well.

## Limitations

The study was conducted in one district, the Lusaka district of Zambia. The sample size reflects the few people working in Zambia on this issue. Conducting interviews on this sensitive topic was not easy as evidenced by the withdrawal of one researcher from the study even after participating in the interviews. In addition, the perspective of the researchers is only one part of the picture, but an important perspective to begin with. We believe that the researchers’ views need to be complemented with those of the adolescents who have participated in PAC clinical and behavioural studies. These situations may limit generalizability of the findings outside the Zambian context, though similar challenges have been documented elsewhere (Gipson et al., 2011; Hess, 2006; Ringheim, 1999). Even though wide generalizability was not the intention, the rich description of phenomena (researcher’s experiences of conducting PAC research in Zambia) and the use of a multi-disciplinary team (with expertise in PAC research, bioethics, law, public health and anthropology) in the study design, data analysis and writing enriched the process as the authors were able to draw on and collate inputs from various professional areas. We believe this study provides a valuable contribution to the knowledge base on ethics challenges experienced by researchers conducting research on post-abortion care with adolescents.

## Conclusion

This study documented ethical challenges related to the conduct of PAC research involving adolescents in Zambia and strategies currently being implemented by researchers to address some of the challenges. Additional stakeholder engagement and discussions to address concerns around when it is appropriate to conduct such research in Zambia and employ some of the above strategies would be beneficial. The development of strategies to enable dissemination to adolescents, families, communities and institutions of information about the legal grounds for abortion in Zambia and the nature of the public health challenge is also likely to be constructive and most effective when done in partnership with public institutions. In addition, it would be important more generally to provide training to researchers in Zambia on acceptable strategies for collecting and disseminating sensitive information.
